# The repeatability of corneal topography measurements in severe Dry Eye disease

**DOI:** 10.1186/s12886-022-02534-4

**Published:** 2022-07-15

**Authors:** Soner Guven

**Affiliations:** Department of Ophthalmology, Kayseri City Hospital, Mevlana mh. Tamer cd. 5/14 Talas, Kayseri, Turkey

**Keywords:** Corneal topography, Repeatability, Pentacam, Severe dry eye disease

## Abstract

**Background:**

To determine the repeatability of corneal topography measurements in severe dry eye disease (DED). A comparison of corneal topography parameters between severe DED and healthy subjects was a secondary goal of this study.

**Methods:**

Sixty-nine patients with severe DED and 46 healthy subjects were enrolled in the study. All participants underwent repeated corneal topography measurements with Pentacam (Oculus, Germany) within a half hour time. Both eyes of the participants were used in statistical analysis. A further subcategorization of severe DED patients was performed according to Ocular Surface Disease Index (OSDI) scores: 32–50, 51–70 and 71–100. The repeatability of corneal parameters was assessed with correlation coefficients (CC).

**Results:**

The mean age of dry eye patients and healthy subjects were 40.8 ± 13.2 (17–66) and 39.8 ± 8.2 (18–61) years (*p*:0.604) respectively. No significant differences were found between severe DED and control groups according to analysed corneal parameters in both eyes (*p*:>0.05). All CCs were greater than 0.9 in severe DED group (*p*:<0.001). All CCs were also greater than 0.9 in severe DED patients among different OSDI groups (*p*:<0.001).

**Conclusions:**

Corneal topography measurements are highly repeatable in severe DED with Pentacam. This is the first report about this topic. Nonetheless, further studies are needed with different topography devices for validation.

## Background

Accurate repeated measurements of an ophthalmic device are crucial to obtain a reliable data in ophthalmology practice. Owing to the leading amount of refractive power is from cornea, corneal topographic data is very useful in cataract and refractive surgery, contact lens fitting, keratoconus, and glaucoma. Pentacam (Oculus, Inc., Germany) is one of the mostly used corneal topography devices which uses Scheimpflug camera. Pentacam tended to measure consistent pachymetry measurements especially in central cornea [1].

“Dry eye disease (DED) is a multifactorial abnormality of the ocular surface with loss of homeostasis of the tear film and accompanied by ocular symptoms. The tear film instability and hyper-osmolarity, ocular surface inflammation and damage, and neuro-sensory abnormalities play etiological roles.” [2]. DED is a globally prevalent disorder (5–50%) affecting older subjects and females more frequently [3]. Precise corneal topography measurements are also essential for their co-existing eye diseases in DED patients.

It has been postulated that Scheimpflug device includes the precorneal tear film layer in measuring corneal thickness [4]. Previous studies showed that Pentacam was repeatable in both healthy and dry eyes [1, 5, 6]. However, a study focusing on the change profile of repeated Pentacam measurements in severe dry eye patients is lacking.

The primary goal of this current study is to explore the repeatability of the corneal topography measurements using the Pentacam in severe dry eye patients. A secondary aim is to compare the corneal topographic measurements in severe dry eye patients with healthy subjects.

## Methods

### Study population

This is a cross-sectional one centre study. All subjects gave verbal consent to participate to this study. The study adhered to the Tenets of Helsinki Declaration. Study protocol was approved by the Clinical Investigations Ethics Committee of Kayseri City Hospital, 2020/251). Adequate sample size for repeated measures was computed as follows:$\frac{1.96\times Sw}{\sqrt{2n\times(m-1)}}=0.2\times Sw=\frac{{1.96}^2}{2\times{0.2}^2\times2}=24$; where S_w_ represents the standard error of the within subject standard deviation; n, the number of subjects and m, the number of measurements per subject when set the confidence interval 20% either side of the estimate of Sw [7]. An additional 20 subjects per each group was included for possible dropouts which gave the final enrolment target of 88 subjects across both groups.

Ophthalmic examination including best corrected visual acuity (BCVA), spheric equivalent, intraocular pressure, Schirmer test with topical anaesthesia, tear break-up time (TBUT), corneal and conjunctival fluorescein staining (CFS) were performed to all participants. A sterile fluorescein strip was placed to outer one third of the lower eyelid fornix. The time interval between the last blink and the first spot seen in precorneal surface under cobalt blue light in seconds was recorded as TBUT. CFS was assessed with fluorescein instillation under cobalt blue according to grading scheme of Oxford and National Eye Institute/Industry Workshop scales respectively [8, 9]. Afterwards, the wetting of the Schirmer test strip located in lower eyelid was measured in millimetres after instillation of proparacaine 0.5% for Schirmer test. Detailed slit-lamp examination of the eyelashes, eyelid margins and meibomian gland orifices with expression of meibomian secretion were performed to rule out any dysfunction of the meibomian glands. A foamy tear film was also considered as a sign for meibomian gland dysfunction (MGD). Ocular surface disease index (OSDI) questionnaire was used to determine subjective discomfort of participants in which scores ranging from 0 (no symptoms) to 100 (worst) [10]. A further subcategorization of severe DED patients were performed according to OSDI scores: 32–50, 51–70 and 71–100. All diagnostic tests were performed 1 day prior to corneal topography to achieve more reliable measurements. Treatment regimen of the patients were also recorded. Most patients were on loteprednol etabonate %0.5 (Lotemax, Bausch Lomb, USA) and artificial tear drops treatment. There were no patients using a topical cyclosporine treatment or having a punctal plug at the time of the study. All participants were instructed to stop all their eye drops at least for 1 day prior to topography examination.

### Scheimpflug imaging

The images of both eyes of each participant were captured after several blinks by an experienced technician who was unaware to the nature of the study design. All scans were performed at the same room with stable humidity and temperature between 10am-2pm to avoid diurnal variations. After headrest position with eyes fixated on an internal target, at least three consecutive high acquisition quality (> 90%) images were obtained at a time. Triplet scans were repeated from each participant after a half hour rest period. Automatic release with 25-image mode was chosen. Flat (K1), steep (K2) and maximum keratometry (Kmax), apical and thinnest corneal thickness (TCT), corneal volume (CV), anterior chamber volume (ACV), corneal diameter and anterior chamber depth (ACD) were recorded from Pentacam. Right and left eyes of the subjects were used separately in analysis to avoid “double-organ bias”. A further cross-check of the results has been achieved by another colleague (S.A) who was masked to the goal of the study to avoid data interpretation bias.

### Inclusion and exclusion criteria

The patients who described dry eye symptoms for at least 3 months, (2) Schirmer result of < 3 mm, (3) TBUT of < 3 s, (4) OSDI score > 32 were enrolled in this study. In case of discordance between OSDI and CFS; a flowchart was used to establish severe DED which was described elsewhere. [11] A further subtype categorization of severe DED was recorded according to the presence of MGD (evaporative type). Subjects with MGD was grouped as mixed type severe DED while subjects without MGD was defined as aqueous deficient type severe DED. Schirmer result of > 10 mm, (2) TBUT > 10 s, (3) OSDI score < 12 were criteria for healthy subjects. Previous history of ocular surgery or ocular trauma, contact lens wear, glaucoma, any type of external ocular surface disease were excluded.

### Statistical analysis

The percentage (%) and number (n) were used for frequency values. The mean SD with range (min-max) were used to present descriptive statistics. The normality of the variables was explored by both Shapiro-Wilk test and Q-Q plots and histograms. Age comparisons between groups were explored by Student’s t test while categorical variables between groups were analysed by chi-square test. General estimating equations (GEEs) were used to account for the effects of bilateral eyes of the same subject on the dependent variables. Correlation coefficients (CC) found in GEEs models were used to assess the repeatability of the topography measurements in each group. CC values greater than 90% was defined as excellent repeatability [12]. All statistical tests were 2-sided and a p value less than 0.05 was accepted as statistically significant.

## Results

A total of 115 participants (69 patients and 46 healthy subjects) met the criteria and included in the analysis. The mean age of dry eye patients and healthy subjects were 40.8 ± 13.2 (17–66) and 39.8 ± 8.2 (18–61) years (p:0.604) respectively. Most dry eye patients in this study were the patients who were referred to ophthalmology outpatient clinics from rheumatologists with a suspected collagen vascular disease (Lupus erythematosus, systemic scleroderma, rheumatoid arthritis, Sjogren syndrome and mixed connective tissue disorders). Most patients were suspected to have rheumatoid arthritis and/or Sjogren syndrome. Several dry eye patients had accompanying diabetes mellitus. No patients were on systemic treatment during the study period. No established specific aetiologies (Graft versus host disease, limbal stem cell deficiency, neurotrophic keratopathy etc.) were evident at the time of the study. Baseline characteristics of the participants were shown in Table 1. All patients were mixed type DED. The two groups did not statistically differ according to analysed topographic parameters (*p*:>0.05) (Table 2). Most of the CC of repeated corneal topography measurements were greater than 0.8 in both groups (Table 3). All CCs were also greater than 0.9 in severe DED patients among different OSDI groups (*p*:<0.001) (Table 4).

**Table 1:** Demographics and clinical characteristics of groups (n:230)

	Dry Eye patients (n:138)	Control subjects (n:92)	p
Sex- female	39 (56.5%)	24 (52.2%)	0.646^*^
Age, years	40.8 ± 13.2	39.8 ± 8.2	0.604^†^
BCVA, Snellen	0.91 ± 0.1	0.94 ± 0.1	0.071^‡^
SE, D	-0.35 ± 0.3	-0.38 ± 0.3	0.433^‡^
IOP, mmHg	14.9 ± 3	14.0 ± 2.7	0.295^‡^
TBUT, seconds	1.32 ± 0.8	15.2 ± 3.2	< 0.001^‡^
Schirmer test, mm/5 min	0.06 ± 0.3	25.7 ± 6.3	< 0.001^‡^
Corneal fluorescein staining^a^	5.89 ± 2.1	0.24 ± 0.4	< 0.001^‡^
Conjunctival fluorescein staining^b^	1.71 ± 1.1	0.2 ± 0.4	< 0.001^‡^
OSDI score	61.62 ± 15	4.21 ± 2.8	< 0.001^‡^

Data are presented as mean ± standard deviation or number (%), where appropriate.

*BCVA* Best corrected visual acuity; *SE* Spheric equivalent; *D* Diopter; *IOP* Intraocular pressure; *TBUT* Tear break-up time; *OSDI* Ocular surface disease index.

^a^NEI/industry-recommended scale (0: no staining, 15: severe staining)

^b^Oxford staining scale 0 to 5 (0: no staining, 5: severe staining)

^*^ Chi-square test

^†^ Independent Student’s t test

^‡^ General Estimating Equations

**Table 2:** Comparison of the Pentacam measurements between dry eye patients and healthy subjects (n:230)

	Dry Eye patients (n:138)	Control subjects (n:92)	p^*^
	**Mean ± SD**	**Mean ± SD**	
K1, diopter	42.93 ± 1.53	42.95 ± 1.44	0.95
K2, diopter	43.99 ± 1.64	43.93 ± 1.49	0.85
Apical pachymeter, micrometer	523.45 ± 52.4	527.98 ± 29.11	0.49
Thinnest pachymeter, micrometer	522.26 ± 32.31	523.18 ± 28.69	0.87
Kmax, diopter	44.82 ± 1.71	44.71 ± 1.61	0.72
Corneal volume, mm^3^	58.72 ± 3.2	58.58 ± 3.2	0.81
Corneal diameter, mm	11.85 ± 0.4	11.86 ± 0.4	0.86
Anterior chamber volume, mm^3^	157.58 ± 42.6	161.3 ± 33.6	0.60
Anterior chamber depth, mm	2.79 ± 0.4	2.87 ± 0.3	0.26

^*^General Estimating Equations

**Table 3:** Repeatability of measurements using the Pentacam in dry and healthy eyes (n: 230)

	Dry Eye patients (n:138)		P*	Control subjects (n:92)		P*
	**Mean (min-max)**	**Model Coefficient (95% Wald CI)**		**Mean (min-max)**	**Model Coefficient (95% Wald CI)**	
K1, D	42.92 (39.4–46.6)	0.986 (0.967–1.005)	< 0.001	42.94 (40.1–46.9)	0.977 (0.950–1.004)	< 0.001
K2, D	43.99 (39.9–47.7)	0.980 (0.962–0.998)	< 0.001	43.94 (40.4–47.3)	0.987 (0.962–1.013)	< 0.001
Apical pachymeter, micrometer	527.25 (429–604)	0.981 (0.953–1.008)	< 0.001	528.48 (474–595)	0.961 (0.913–1.010)	< 0.001
Thinnest pachymeter, micrometer	522.2 (422–601)	0.974 (0.944–1.005)	< 0.001	523.18 (469–592)	0.988 (0.972–1.007)	< 0.001
Kmax, D	44.84 (40.3–48.5)	0.901 (0.845–0.957)	< 0.001	44.71 (40.8–48.6)	0.948 (0.877–1.020)	< 0.001
Corneal volume, mm^3^	58.73 (50–67)	0.981 (0.938–1.003)	< 0.001	58.57 (51.8–66.8)	0.987 (0.969–1.007)	< 0.001
Corneal diameter, mm	11.85 (10.2–12.7)	0.836 (0.593–1.008)	< 0.001	11.86 (11-13.1)	0.955 (0.919–0.992)	< 0.001
Anterior chamber volume, mm^3^	157.61 (73–260)	0.988 (0.964–1.012)	< 0.001	161.3 (63.6–234)	0.892 (0.736–1.036)	< 0.001
Anterior chamber depth, mm	2.79 (1.07–3.98)	0.983 (0.817–1.009)	< 0.001	2.86 (2.13–3.56)	0.923 (0.874–0.972)	< 0.001

*General Estimating Equations; *CI* confidence interval; *D* diopter

**Table 4:** Repeatability of Measurements Using the Pentacam in Dry Eyes Among Different OSDI Groups (n:138)

	OSDI scores					
	**32–50** **(n: 30)**		**51–70** **(n: 72)**		**71–100** **(n: 36)**	
	**Model Coefficient (95% Wald CI)**	P*	**Model Coefficient (95% Wald CI)**	P*	**Model Coefficient (95% Wald CI)**	P*
K1, D	0.987 (0.968–1.005)	< 0.001	0.978 (0.938–1.018)	< 0.001	0.978 (0.950–1.007)	< 0.001
K2, D	0.980 (0.950–1.010)	< 0.001	0.977 (0.941–1.023)	< 0.001	0.994 (0.967–1.021)	< 0.001
Apical pachymeter, micrometer	0.988 (0.970–1.006)	< 0.001	0.982 (0.949–1.016)	< 0.001	0.961 (0.871–1.052)	< 0.001
Thinnest pachymeter, micrometer	0.985 (0.964–1.006)	< 0.001	0.971 (0.934–1.008)	< 0.001	0.988 (0.975–1.001)	< 0.001
Kmax, D	0.947 (0.887–1.006)	< 0.001	0.868 (0.765–0.971)	< 0.001	0.958 (0.885–1.030)	< 0.001
Corneal volume, mm^3^	0.942 (0.808–1.076)	< 0.001	0.984 (0.961–1.007)	< 0.001	0.922 (0.827–1.017)	< 0.001
Corneal diameter, mm	0.970 (0.920–1.020)	< 0.001	0.974 (0.933–1.015)	< 0.001	0.962 (0.926–0.999)	< 0.001
Anterior chamber volume, mm^3^	0.984 (0.959–1.009)	< 0.001	0.996 (0.987–1.005)	< 0.001	0.957 (0.930–0.984)	< 0.001
Anterior chamber depth, mm	0.992 (0.980–1.004)	< 0.001	0.966 (0.913–1.018)	< 0.001	0.993 (0.937–1.050)	< 0.001

*General Estimating Equations; *OSDI* Ocular surface disease index; *CI* confidence interval; *D* diopter

## Discussion

In contrast to expected tear-film instability accompanied with possible distorted ocular surface, this study demonstrated excellent agreement in corneal topography measurements between severe DED patients and healthy controls. All ICCs were greater than 0.9 in severe DED patients. Excellent repeatability of the corneal topography measurements also persisted even in patients with highest OSDI scores (OSDI score 71–100). Surprisingly, ACD and ACV ICCs were found to be lower than 0.9 in healthy subjects. This result could be attributed to lower sample size in healthy subjects. This current study also showed no difference in Scheimpflug based corneal topography parameters between severe DED patients and control subjects.

In previous reports the investigators assessed the repeatability of central corneal thickness (CCC) by Pentacam and corneal topography measurements by Sirius devices [5, 6]. Lee et al. reported that CCT undulated in dry eyes and found that ICC was lower in dry eyes than healthy eyes by Pentacam [5]. By contrast, Dogan et al. reported excellent repeatability both in their dry eye patients and healthy controls with Sirius device [6]. Dogan et al. also speculated their excellent repeatability results could be arisen from the high speed image capacity of the device which might be faster than TBUT of the dry eye patients [6]. Similarly, Pentacam has also high-speed image acquisition capacity which lasts less than 2 s. This hypothesis might have also led to high repeatability results in this cohort. Additionally, precorneal tear-film routinely might not be detected by Pentacam unless fluorescein instillation [13]. Exclusion of tear-film layer in Pentacam images might have a role in high ICCs observed in all corneal parameters of severe DED patients of this current report. In this manner, Lee et al. did not observe a rise in CCT measurements after tear supplement instillations in their cohort [5]. However, in contrast to this current study neither of the aforementioned studies included severe DED patients [5, 6].

One possible explanation for the high repeatability in this cohort might be due to relatively low CFS (5.89 ± 2.1 (1–10)) scores observed in dry eye patients indicating that ocular surface damage was not particularly evident. Tough both Dogan and Lee et al. targeted the similar goal, the repeatability of corneal topography measurements in dry corneas, neither of them mentioned the mean scores of CFS to compare with this report [5, 6].

Tear film staining with fluorescein was associated with increased backscattering of light at the anterior corneal surface in rotating Scheimpflug technique which might lead to measurement errors due to brighter signal detection [14]. Supporting the effect of florescent tear film on Scheimpflug images, overall tear film distribution tended to differ from normal corneas in dry corneas especially with a superior corneal thickening trend [13]. The tear film was not stained with fluorescein in this present study. Although, all patients were severe DED in this study, no significant changes were observed in Scheimpflug images compared to healthy subjects. It is difficult to distinguish the source of measurement changes seen in DED in Scheimpflug images either from the fluorescein effect or tear film abnormalities or both. Further studies are needed to conclude.

Meyer et al. investigated the density of corneal layers and CCT in dry eye patients with Scheimpflug photographs [15]. Corneal density was found to be significantly reduced in all layers (epithelium, Bowman and Descemet membrane) compared to healthy controls [15]. CCT was also found to be reduced in dry eye patients but the significance level was relatively debatable as a p value of 0.0495 [15]. It is important to note that the investigators assessed only the Scheimpflug photographs instead of Pentacam topographer in contrast to present study [15]. The authors of a recent study comparing Pentacam and anterior segment optic coherence tomography (ASOCT) reported that Pentacam provided significantly higher CCT and TCT values in severe DED group compared to ASOCT after adjusting age and sex [16]. The investigators also speculated that difference in severe DED group could have been arisen from the fact that Pentacam was less susceptible to tears [16]. Although Fujimoto et al. included severe DED patients, the severe DED patient profile was different from this present study in regard to DED type (higher Schirmer values representing higher aqueous deficiency, lower symptomology (different questionnaire) and lower CFS scores) [16].

Parallel to this study, Zemova et al. found no significant interaction between DED and topographic/tomographic measurements in keratoconus patients [17]. Consistent with this present study, the repeatability of corneal thickness measurements were found to be high both in normal, post-laser in situ keratomileuses and keratoconus eyes [1]. Recently, Cicek et al. reported that higher limits of repeatability in K1, K2 and Kmax values in keratoconus patients after cross-linking treatment with Pentacam [18]. However, all computed corneal topography parameter ICCs were reported to have over 0.95 both in CXL and naïve keratoconus patients in the same report [18]. These results were also parallel to this current study.

This study has some limitations. Patient selection and disease categorization bias are subject to DED investigations [19]. The reported symptom severity may not always reflect the clinical signs of the disease and there may be a proportion of patients who have conflicting signs and symptoms. Certain subtypes of DED (e.g., MGD) might be more asymptomatic compared to other subtypes and may not correlate with the severity of the ocular surface damage [20]. Correspondingly, CFS scores do not necessarily correlate with OSDI scores. A similar repeatability analysis for high score CFS patients regardless of their OSDI scores might give additional information. Unfortunately, this analysis could not be performed due to having patients with relatively lower CFS scores (maximum 8) in this report. A selection bias in recruiting dry eye patients for the study might have been occurred by not including the most severe forms of DED (with higher CFS scores) in the study. Furthermore, no strict criteria have been described for severe DED even in DEWS 2 report [19]. Thus, possible patient selection bias and DED categorization bias may have also theoretically occurred in this report. There are various diagnostics criteria to evaluate DED such as conjunctival staining, Schirmer test, blurred vision, filamentary keratitis, tear hyperosmolarity, impression cytology, blepharospasm, meibomian gland disease or eyelid inflammation, TBUT, corneal sensitivity, and inflammatory markers (HLA-DR, MMP9, cytokines etc.) [11]. One novel diagnostic test or criteria which was not used as an inclusion criterion might have changed both patient selection and DED severity [19]. Tear osmolarity comparisons were also lacking in this report. Most patients studied in this report were mixed type DED. Although, the most common subtype of DED is mixed type, the results of this current study should be interpreted cautiously due to reporting only one subtype of severe DED. The repeated Pentacam measurements of different subtypes of severe DED other than mixed type (aqueous deficient or evaporative) might not be parallel to this report. However, subtype identification of the DED might only be important in aiding the management of this condition but it is not the primary goal of this study and perhaps it may be an interesting study topic in further studies.

One centre design is also another limitation of this study. The effect of artificial tear drops on Pentacam measurements in severe DED could also be explored but it was out of scope of this study. Major strength of this study is that it is the preliminary report analysing the repeatability of corneal topography parameters in severe DED. The negative results of this report in this “extreme” type of study population might be useful for clinicians in managing refractive surgery candidates, detecting corneal pathologies, and assessment of the intraocular pressure measurements in glaucoma patients.

## Conclusions

Corneal topography measurements were found to be highly repeatable in severe DED patients with Pentacam device. Nevertheless, further multi-centre studies are essential in severe DED with other anterior segment devices rather than Pentacam to confirm the results of this current report.

## Data Availability

Data are available from the corresponding author on reasonable request.

## Abbreviations

DED: Dry eye disease
GEEs: General estimating equations
MGD: Meibomian gland dysfunction
CC: Correlation coefficient
BCVA: Best corrected visual acuity
TBUT: tear break-up time
CFS: Conjunctival fluorescein staining
OSDI: Ocular surface disease index
TCT: Thinnest corneal thickness
CV: Corneal volume
ACV: Anterior chamber volume
ACD: Anterior chamber depth
CCT: Central corneal thickness
